# Untargeted Metabolomics Reveals the Function of GPRC6A in Amino Acid and Lipid Metabolism in Mice

**DOI:** 10.3390/metabo12090776

**Published:** 2022-08-23

**Authors:** Yumin He, Jingyun Su, Hongrui Gao, Jianzhong Li, Zemeng Feng, Yulong Yin

**Affiliations:** 1 Hunan Provincial Key Laboratory of Animal Nutritional Physiology and Metabolic Process, Key Laboratory of Agro-Ecological Processes in Subtropical Region, Institute of Subtropical Agriculture, Chinese Academy of Sciences, National Engineering Laboratory for Pollution Control and Waste Utilization in Livestock and Poultry Production, Hunan Provincial Engineering Research Canter for Healthy Livestock and Poultry Production, Scientific Observational and Experimental Station of Animal Nutrition and Feed Science in South-Central, Ministry of Agriculture, Changsha 410125, China(; 2 Animal Nutrition and Human Health Laboratory, College of Life Sciences, Hunan Normal University, Changsha 410081, China; 3 College of Veterinary Medicine, Hunan Agricultural University, Changsha 410128, China

**Keywords:** GPRC6A, metabolomics, lipid, amino acids

## Abstract

GPRC6A is an amino acid sensor in the cytomembrane. Despite substantial evidence for the role of GPRC6A in metabolism, the specific effects and mechanism by which this gene acts on metabolic processes are still unresolved. In this study, serum biochemical parameters related to liver and kidney function and serum amino acid levels were determined in GPRC6A wild-type (WT) and knockout (KO) mice. An untargeted serum metabolomics analysis was also conducted for the first time, to the best of our knowledge, to decipher the function of GPRC6A in metabolic processes. GPRC6A was involved in lipid and amino acid metabolism, mainly by affecting liver function. A loss of GPRC6A function may perturb bile acid metabolism, thus leading to abnormal unsaturated fatty acid metabolism. GPRC6A KO may lead to excessive protein breakdown under starvation, and the loss of GPRC6A had a significant effect on phenylalanine metabolism-related pathways. Our metabolomics data provide a novel basis for further functional studies of GPRC6A.

## 1. Introduction

G-protein-coupled receptors (GPCRs) are the largest mammalian membrane receptor family and function as neurotransmitters and hormone receptors. Recent studies have shown that GPCRs can also sense nutrients [1,2]. According to sequence homology, GPCRs are divided into three categories: A, B, and C [3]. Class C GPCRs are characterized by a large extracellular domain containing a nutrient-sensitive Fly Trap domain (VFT) and a Cys-rich domain [3]. GPCRs mainly include the classic neurotransmitter metabotropic glutamate receptor subtype-1 (mGluR1), type 1 taste receptors (T1Rs), G-protein-coupled receptor class C group 6 member A (GPRC6A), and calcium-sensing receptor (CaSR) [2].

GPRC6A was identified in 2004, when it was cloned and expressed from human kidney cDNA. It has a seven-fold transmembrane domain and a long amino terminal domain, which is highly homologous to those of other GPCRs [4]. It shares 34%, 28%, and 24% amino acid sequence homology with CaSR, T1R1, and mGluR1, respectively [4]. In mice, the gene is expressed in the brain, salivary gland, skeletal muscle, heart, lung, spleen, kidney, liver, stomach, testis, and early embryo [5,6,7]. With respect to the subcellular localization, the gene is expressed on the cell membrane [8,9,10]. Alkaline amino acids (l-Arginine, l-Lysine, and l-Ornithine), divalent cations [11,12,13,14], and testosterone [15] are efficient activators of GPRC6A; however, the ability of osteocalcin to effectively activate GPRC6A is still controversial [16,17,18,19].

Many studies have shown that GPRC6A plays an important role in diverse metabolic processes, such as glucose homeostasis [20,21,22,23]. Clemmensen et al. demonstrated that GPRC6A plays an important role in energy metabolism [24], and this was further verified in liver-specific GPRC6A knockout (KO) mice [25]. In addition, GPRC6A plays a role in bone metabolism and acts as a nutrient receptor in the gastrointestinal tract [6,26,27]. Studies have shown that GPRC6A is associated with the progression of adenocarcinoma, inflammation, non-alcoholic fatty liver, and other pathological states and can affect male reproductive ability [14,28,29,30,31]. A study has shown that adipose-specific GPRC6 KO mice are more susceptible to diet-induced obesity by inhibiting lipolysis [32]. In general, GPRC6A plays an important role in metabolism. However, as an amino acid sensor, the mechanism by which GPRC6A mediates amino acid metabolism remains unclear.

Recent advances in equipment and analytical techniques, especially in omics techniques, have provided a basis for expanded research in life sciences [33]. A transcriptomics analysis revealed the molecular mechanism by which GPRC6A tunes metabolic function in the liver [25]. However, metabolomics analyses of the function of GPRC6A are lacking. In this study, biochemical and serum metabolomics analyses were performed to elucidate the effect of a GPRC6A deficiency on metabolic processes.

## 2. Materials and Methods

### 2.1. Animals and Diets

Global GPRC6A^−/−^ C57BL/6 mice were obtained from the Shanghai Model Organisms Center (Shanghai, China) and backcrossed to WT C57BL/6 mice for at least six generations. GPRC6A^−/−^ and GPRC6A^+/+^ littermates used in experiments were generated by heterozygote parents to eliminate effects of genetic background. All animal procedures were approved by the Animal Ethics Committee of the Institute of Subtropical Agriculture, Chinese Academy of Sciences.

We designed two experiments. In Experiment 1, GPRC6A WT and KO mice were fed standard chow for rodents from 3 weeks to 55 weeks. At 8 weeks, a portion of WT and KO mice were sacrificed to collect samples, which were assigned to WT-young (*n* = 8) and KO-young (*n* = 7) groups. At 55 weeks, the remaining mice were sacrificed for sample collection, which were allocated to WT-old (*n* = 8) and KO-old (*n* = 7) groups.

In Experiment 2, GPRC6A WT and KO mice were weaned at 21 days of age, fed standard chow for rodents, and divided into four groups, WT–NP (*n* = 11), KO–NP (*n* =10), WT–LP (*n* = 10), and KO–LP (*n* = 9), and fed two kinds of feed differing in protein levels. The formulations are shown in Table 1.

All mice were housed at 23 ± 2 °C, 55 ± 5% humidity, with a 12:12 h light–dark cycle and with food and demineralized water ad libitum.

### 2.2. Hematoxylin–Eosin (HE) Staining

The liver and kidney were fixed with 4% formaldehyde (Servicebio, Cat#G1101, Wuhan) and sliced. Hematoxylin and eosin (HE) staining was performed for morphological observations using a Zeiss 902 transmission electron microscope.

### 2.3. Blood Collection and Serum Separation

In mice fed a standard chow from 3–55 weeks, feeds were removed, and blood was then immediately taken from the ocular margin vein. For mice fed diets with different protein levels, the mice were fasted for 8 h before blood was taken. Blood samples were placed at 25 °C for 1 h, then centrifuged at 300 rpm (4 °C) for 10 min, and the supernatants were collected for biochemical detection and metabolite extraction.

### 2.4. Biochemical Parameter Determination

The serum samples were diluted two times in saline solution and then used for biochemical determination. Serum total protein (TP, Roche-03183734190), albumin (ALB, Roche-03183688122), blood urea nitrogen (BUN, Roche-20766682322), AST (Roche- 20764949322), ALT (Roche-20764957322), uric acid (UA, Roche-03183807190), triglyceride (TG, Roche-20767107322), total bile acid (TBA, Roche-11732650001), cholesterol (CHOL, Roche-03039773190), lipase (Roche-03029590322), and glucose (Roche-04404483190) were determined using kits from Roche Diagnostics (Division of Hoffman-la Roche Limited, Montreal, QC, Canada) and the Hitachi 7150 autoanalyzer (Hitachi Medical, Tokyo, Japan), according to the manufacturers’ instructions.

### 2.5. Metabolite Extraction

Each sample (50 μL) was transferred to an EP tube. After the addition of 200 μL of extract solution (acetonitrile: methanol = 1:1, containing isotopically labelled internal standard mixture), the samples were vortexed for 30 s, sonicated for 10 min in an ice-water bath, and incubated for 1 h at −40 °C to precipitate proteins. Then, the sample was centrifuged at 12,000 rpm (RCF = 13,800× *g*, R = 8.6 cm) for 15 min at 4 °C. The resulting supernatant was transferred to a fresh glass vial for analysis. A quality control (QC) sample was prepared by mixing an equal aliquot of the supernatants from all of the samples.

### 2.6. LC-MS/MS

LC-MS/MS analyses were performed by using a UHPLC system (Vanquish, Thermo Fisher Scientific, Waltham, MA, USA) equipped with a UPLC BEH Amide column (2.1 mm × 100 mm, 1.7 μm) coupled to a Q Exactive HFX mass spectrometer (Orbitrap MS, Thermo) according to a published protocol [34]. The mobile phase consisted of 25 mmol/L ammonium acetate and 25 mmol/L ammonia hydroxide blended in water (pH = 9.75) (a) and acetonitrile (b). The autosampler temperature was set at 4 °C, and 3 μL samples were injected. The QE HFX mass spectrometer was used to acquire MS/MS spectra in information-dependent acquisition (IDA) mode using acquisition software (Xcalibur, Thermo). The acquisition software continuously evaluates the full-scan MS spectra in this mode. The ESI source conditions were as follows: sheath gas flow rate 30 Arb, Aux gas flow rate 25 Arb, capillary temperature 350 °C, full MS resolution 60,000, MS/MS resolution 7500, collision energy 10/30/60 in NCE mode, spray voltage 3.6 kV (positive) or −3.2 kV (negative).

### 2.7. Data Preprocessing and Annotation

Raw LC-MS/MS data were converted to the mzXML format using ProteoWizard and processed using a program that was developed using R based on XCMS for peak detection, extraction, alignment, and integration. Then, an MS2 database (BiotreeDB) was applied for metabolite annotation. The cutoff for annotation was set at 0.3.

PCA and OPLS-DA were performed using MetaboAnalyst 5.0. PCA and OPLS-DA were performed based on the normalized peak area of the metabolites in each sample. The annotated data were normalized by the sum of values, subjected to cube root transformation, mean-centered, and divided by the standard deviation of each variable. The variable importance in projection (VIP) values for metabolites were calculated by OPLS-DA. Permutation tests were performed with 100 replicates. R commands are provided in the Supplementary Materials. Values of *p* < 0.05, fold change >1.5, and VIP > 1 were used as thresholds to identify significant differential metabolites.

### 2.8. KEGG Pathway Analysis

Significant differential metabolites under NEG or POS mode were combined for a pathway enrichment analysis based on KEGG human metabolic pathways (October 2019). Only metabolite sets containing at least two entries were used. The enrichment ratio was computed by Hits/Expected, where hits = observed hits and expected = expected hits. A pathway with a value of *p* < 0.05 was considered significant.

To evaluate body weight, feed intake, amino acid concentrations, and biochemical parameters of the serum, abnormal values were excluded (quartile). Data were analyzed by two-way ANOVA and post hoc Student’s *t*-tests. A *p* value <0.05 was considered significant.

## 3. Results

### 3.1. Effect of Dietary Protein on GPRC6A^+/+^ and GPRC6A^−/−^ Mice

GPRC6A^+/+^ and GPRC6A^−/−^ mice were fed either a normal-protein (NP; 20% dietary protein) diet or a low-protein (LP; 10% dietary protein) diet for 3 to 8 weeks (see Table 1 for diet formulations). Genotype had a minor influence on body weight and cumulative feed intake during this period (Figure S1). The protein level affected the body weight of mice 4–5 weeks after birth; however, this difference disappeared thereafter. Further, hematoxylin–eosin (HE) staining of the liver showed that the loss of GPRC6A led to a marked dilatation of hepatic sinuses and uneven chromatin, especially when dietary protein levels were insufficient (Figure S1), while the histomorphology of the kidney was semblable. When GPRC6A^+/+^ and GPRC6A^−/−^ mice were fed a standard chow for rodents up to 55 weeks of age, GPRC6A^−/−^ mice showed a significantly higher body weight than that of GPRC6A^+/+^ mice (data not shown).

### 3.2. Loss of GPRC6A Altered Serum Biochemical Parameters

**Figure 1:**
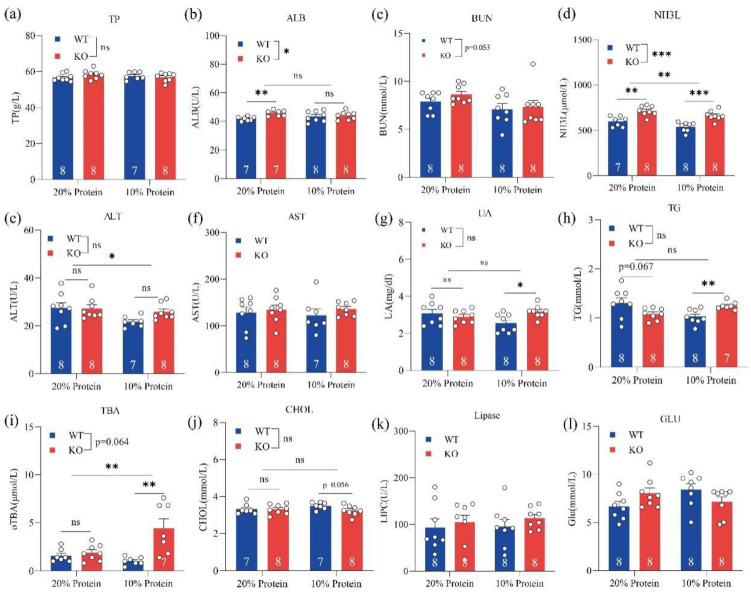
Effects of a GPRC6A deficiency on serum biochemical parameters in mice fed diets with different protein levels. Data are presented as means ± SE; ns represents not significant; * *p* < 0.05, ** *p* < 0.01, *** *p* < 0.001, *n* = 7–8. (**a**) TP (Total protein), (**b**) ALB (Albumin), (**c**) BUN (Blood urea nitrogen), (**d**) NH3L (Blood ammonia), (**e**) ALT (Alanine aminotransferase), (**f**) AST (Aspartate aminotransferase), (**g**) UA (Uric acid), (**h**) TG (Triglycerides), (**i**) TBA (Total bile acid), (**j**) CHOL (Cholesterol), (**k**) Lipase, (**l**) GLU (Glucose).

To investigate how the loss of GPRC6A function affects serum biochemical parameters and metabolic processes, we designed two experiments. In one experiment, GPRC6A wild-type (WT) and KO mice were fed 20% or 10% dietary protein from weeks 3 to 8. In the other experiment, GPRC6A WT and KO mice were fed a standard chow for rodents from weeks 8 to 55. Serum biochemical parameters related to protein metabolism, lipid metabolism, glucose metabolism, and hepatic and renal function were measured. In mice fed different dietary protein levels, we found that genotypes and dietary protein levels had minor effects on serum TP (total protein), BUN (blood urea nitrogen), AST (aspartate aminotransferase), UA (uric acid), TG (triglyceride), CHOL (cholesterol), lipase, and GLU (glucose) levels (Figure 1). However, NH3L (blood ammonia) levels were significantly higher in GPRC6A^−/−^ mice than those in GPRC6A^+/+^ mice. In mice fed an LP diet, the serum TBA (total bile acid) was obviously higher than that in mice fed an NP diet.

**Figure 2:**
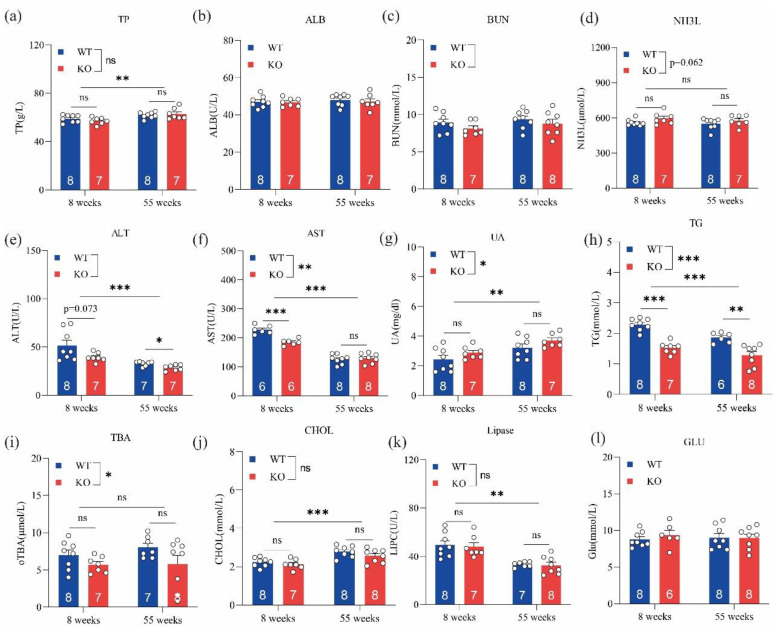
Effects of GPRC6A deletion on serum biochemical parameters in mice at 8 and 55 weeks. Data are presented as means ± SE; ns represents not significant; * *p* < 0.05, ** *p* < 0.01, *** *p* < 0.001, *n =* 7–8. (**a**) TP (Total protein), (**b**) ALB (Albumin), (**c**) BUN (Blood urea nitrogen), (**d**) NH3L (Blood ammonia), (**e**) ALT (Alanine aminotransferase), (**f**) AST (Aspartate aminotransferase), (**g**) UA (Uric acid), (**h**) TG (Triglycerides), (**i**) TBA (Total bile acid), (**j**) CHOL (Cholesterol), (**k**) Lipase, (**l**) GLU (Glucose).

Biochemical parameters, including TP, ALT, AST, UA, TG, lipase, and CHOL, were sensitive to age, with significantly different levels between young and old mice (i.e., 8 and 55 weeks) (Figure 2). ALT, AST, TG, and TBA levels in GPRC6A^+/+^ mice were significantly higher than those of GPRC6A^−/−^ mice; however, UA levels in GPRC6A^−/−^ mice were significantly higher than those in GPRC6A^+/+^ mice. These results suggested that the loss of GPRC6A exerts adverse effects on liver and kidney function, and these effects may regulate lipid and amino acid metabolism.

### 3.3. Loss of GPRC6A Altered Free Amino Acid Levels in Mice

As shown in Table 2, in the serum of GPRC6A^−/−^ mice fed 20% dietary protein, Arg, Glu, Thr, Tyr, Met, and Trp concentrations were significantly higher than in GPRC6A^+/+^ mice. When the protein level in feed was 10%, serum Gln and Ala levels in GPRC6A^+/+^ mice were significantly higher than those in GPRC6A^−/−^ mice, while Tyr levels showed the opposite pattern. At 55 weeks of age, there were minor differences in amino acid content between GPRC6A^+/+^ and GPRC6A^−/−^ mice when mice were not fasted (Figure S2).

### 3.4. Metabolomics Screening for Differentially Expressed Metabolites between GPRC6A ^+/+^ and GPRC6A^−/−^ Mice

Metabolomics is usually carried out by combining chromatography and mass spectrometry. Mass spectrometry requires positive and negative ionization of metabolites, resulting in two ion flow spectra, i.e., positive ion (POS) and negative ion (NEG). Under the POS mode, 511 metabolites from over 106 classes were annotated, including 72 glycerophosphocholine metabolites and 84 amino acids, peptides, and analogues. Under the NEG mode, 227 metabolites from over 55 classes were annotated, including 37 amino acids, peptides, and analogues, 19 carbohydrates and carbohydrate conjugates, and 39 fatty acids and conjugates.

**Figure 3:**
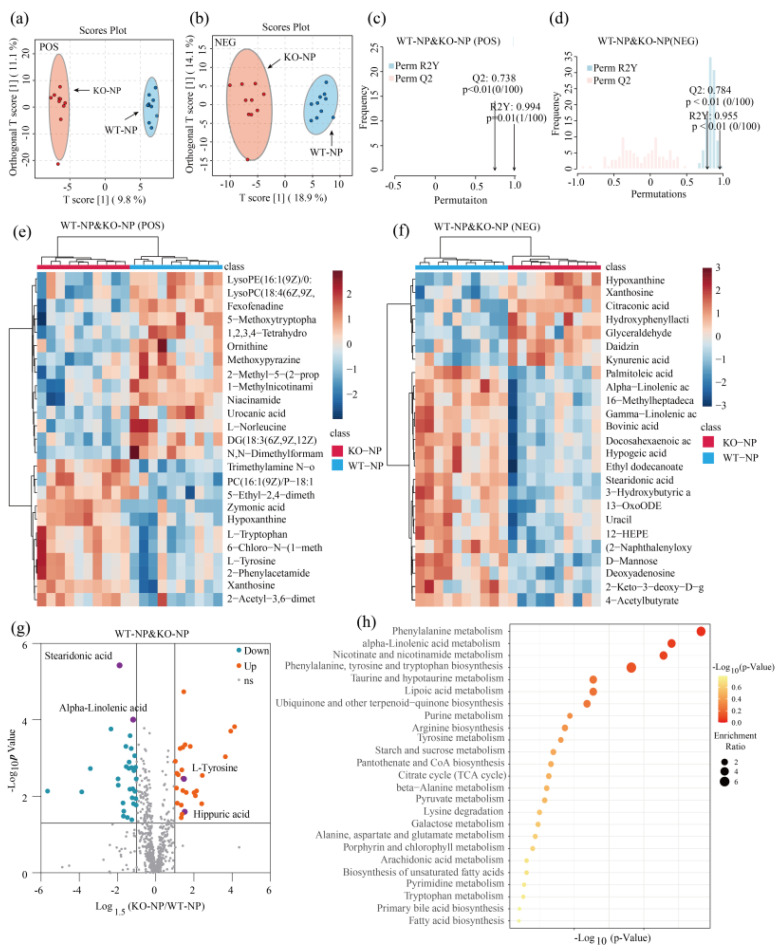
Knockout of GPRC6A altered metabolic profiles of mice fed 20% dietary protein. (**a**,**b**) OPLS–DA score plots showed good separation of clusters between WT–NP and KO–NP under POS or NEG mode. (**c**,**d**) Permutation test verified the reliability of OPLS–DA, with 100 permutations. (**e**,**f**) Heatmap of metabolites with the most significant differences based on *p*-values (Top 25). (**g**) Volcano plot shows the differential metabolites between WT–NP and KO–NP; the vertical axis represents the *p*-value (−log_10_ *p*-value), and the horizontal axis represents the fold change (Log_1.5_FC), where FC is defined as the ratio of levels in KO–NP to WT–NP. (**h**) Overview of enriched metabolite sets (Top 25); the horizontal axis represents the *p*-value (−log_10_ *p*-value); enrichment ratio is the ratio of observed hits to expected hits.

**Figure 4:**
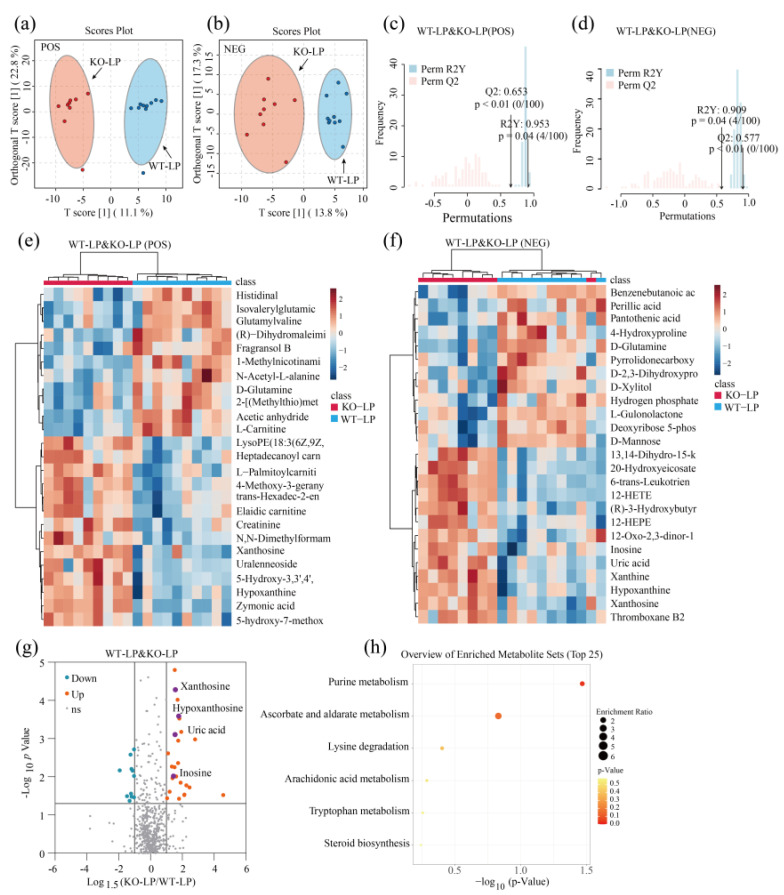
Knockout of GPRC6A altered the metabolic profile of mice fed 10% dietary protein. (**a**) Volcano plot shows the differential metabolites between WT–LP and KO–LP mice; the vertical axis represents the *p*-value (−log_10_ *p*-value), and the horizontal axis represents the fold change (Log_1.5_FC) under NEG mode, where FC is defined as the ratio of KO–LP to WT–LP. (**b**) Heatmap based on *p*-values under NEG mode (Top 25 are presented). (**c**) Volcano plot shows the differential metabolites between WT–NP and KO–NP mice; the vertical axis represents *p*-value (−log_10_ *p*-value), and the horizontal axis represents the fold change (Log_1.5_FC) under POS mode, where FC is defined as the ratio of KO–LP to WT–LP. (**d**) Heatmap based on *p*-values under POS mode (Top 25 are presented). (**e**,**f**) Heatmap of metabolites with the most significant differences based on *p*−values (Top 25). (**g**) Volcano plot shows the differential metabolites between WT−LP and KO−LP; the vertical axis represents the *p*−value (−log_10_ *p*−value), and the horizontal axis represents the fold change (Log1.5FC), where FC is defined as the ratio of levels in KO−LP to WT−LP. (**h**) Overview of enriched metabolite sets (Top 25 are presented); the horizontal axis represents *p*-values (−log_10_ *p*-value); enrichment ratio was defined as the ratio of observed hits to expected hits.

Two-dimensional principal component analysis (PCA) score plots clearly revealed that QC samples aggregated well under both POS and NEG mode, supporting the stability of the metabolomics analysis (Figure S3a,b). We conducted a supervised orthogonal partial least-squares discriminant analysis (OPLS-DA) under POS and NEG ion modes. As shown in Figure 3a,b and Figure 4a,b, significant separation of clusters was detected between GPRC6A WT and KO mice for the same protein level, indicating that the loss of GPRC6A resulted in a significant difference in serum metabolite profiles. To test the reliability of the OPLS-DA results, permutation tests were performed. A shown in Figure 3c, R2Y was 0.994 (*p* = 0.01), and Q2 was 0.783 (*p* < 0.01), indicating that the OPLS-DA model of WT–NP and KO–NP mice under POS mode can be well-explained. Similarly, permutation tests indicated that models of WT–NP and KO–NP under NEG mode were not overfitted (Figure 3d). To preliminarily evaluate metabolite changes, we compared annotated metabolites between groups by Student’s *t*-tests; metabolites with the most significant differences based on *p*-values are represented in heatmaps (Figure 3e,f and Figure 4e,f), revealing that samples from the same treatment group form distinct clusters.

In this study, the criteria to identify a differential metabolite were as follows: *p* < 0.05, |*Log*_1.5_*FC*| > 1, and VIP > 1. A total of 56 metabolites differed significantly between WT–NP and KO–NP (combining metabolites under POS and NEG modes), as shown in a volcano plot in Figure 3g, among which 25 metabolites were upregulated and 31 were downregulated. As for WT–LP and KO–LP, 25 metabolites were upregulated, and 11 metabolites were downregulated (Figure 4c); detailed parameters are given in Tables S1 and S2.

### 3.5. Pathway Enrichment Analysis Based on the KEGG (Kyoto Encyclopedia of Genes and Genomes) Library

A pathway analysis of multiple metabolites can be more informative than analyses of individual metabolites. In this study, we imported the differential metabolites to the KEGG database to explore related metabolic pathways. Differentially expressed metabolites obtained in POS and NEG modes were combined. In Figure 3h, the top 25 enriched metabolites for WT–NP and KO–NP are displayed. Several metabolic pathways related to amino acid metabolism and lipid metabolism were screened, among which the phenylalanine metabolism pathway differed significantly between the two groups (*p* < 0.05). The differential metabolites related to phenylalanine metabolism were l-tyrosine and hippuric acid (Figure 3g). In regard to lipid metabolism, the most significant metabolic pathway was alpha-linolenic acid metabolism (*p* = 0.06). This differential metabolic pathway mainly involved alpha-linolenic acid and stearidonic acid. Thirty-six differential metabolites between WT–LP and KO–LP were uploaded to the KEGG database to explore related pathways. Only one metabolic pathway related to purine metabolism (Figure 4h) differed significantly between groups (*p* < 0.05), and this pathway involved xanthosine, hypoxanthine, inosine, and UA (Figure 4g).

## 4. Discussion

The function of GPRC6A in metabolism has been a long-standing issue. There is evidence that GPRC6A plays an important role in metabolism [10]. GPRC6A is involved in lipid metabolism in mice, and the loss of GPRC6A could promote diet-induced obesity by inhibiting the decomposition of fat [32]. However, the detailed functions of GPRC6A are controversial, particularly its effects on basal glucose in mice [20,35,36]. As an amino acid receptor, few studies have evaluated whether GPRC6A is related to amino acid metabolism in mice. Therefore, in this study, we determined serum biochemical parameters, serum free amino acid levels, and serum metabolomics profiles to explore the impact of GPRC6A on metabolism.

We did not obtain sufficient evidence for an effect of GPRC6A deletion on blood glucose homeostasis in mice. There were no significant differences between WT and KO mice under normal feeding or fasting conditions, indicating that GPRC6A deletion did not affect resting blood glucose in mice. We cannot exclude the possibility that GPRC6A WT and KO mice behave differently in response to external stimuli, such as intraperitoneal glucose injection [25].

HE staining of the liver of 8-week-old mice showed that the loss of function of GPRC6A resulted in an abnormal liver structure, consistent with previous research suggesting that the loss of GPRC6A leads to obesity in mice due to impaired lipolysis [32]. We did not detect liver steatosis. This may be attributed to the normal fat percentage in our diets; the feeding period was only 5 weeks, and a former study has shown that a high-fat diet for 20 weeks induces obesity phenotypes in GPRC6A KO mice [23]. In the normal physiological state without fasting, the loss of GPRC6A led to a decrease in the activity of enzymes secreted by the liver in mouse serum, mainly ALT and AST, similar to aging-related changes. Liver dysfunction has also been observed in mice with liver tissue-specific gene KO [25], consistent with our results. In summary, we believe that GPRC6A deletion influences the integrity of liver function in mice.

We believe that GPRC6A plays a role in lipid metabolism. In both young and old mice without fasting, the serum TG levels of WT mice were significantly higher than those of KO mice, suggesting that KO mice have abnormal lipid metabolism, to a certain extent [37]. However, there is no evidence that the loss of GPRC6A affects lipase activity. In this study, age influenced lipase levels in the blood, while genotype did not. GPRC6A KO mice had lower serum bile acid levels; since the lack of TBA would affect the decomposition efficiency of lipids by lipase, abnormal TBA secretion may disrupt lipid metabolism in GPRC6A KO mice [38]. Our metabolomics analysis indicated that GPRC6A played a significant role in lipid metabolism, mainly by affecting pathways related to alpha-linolenic acid metabolism.

Ammonia produced by the catabolism of various amino acids in the body and the ammonia absorbed by the intestine is transported to the blood; this blood ammonia is transported to the liver, transformed to urea, and excreted through the kidneys [39]. In this study, the NH3L levels of GPRC6A KO mice were significantly higher than those of WT mice in the fasting state. Since exogenous nitrogen was unavailable during fasting, blood ammonia was derived from the catabolism of nitrogen-containing substances in the body. We also found that the levels of some amino acids in KO serum were significantly higher than those in WT mice, which may be explained by the enhanced catabolism of nitrogenous substances in mice under starvation due to the loss of GPRC6A. Studies have shown that under starvation, proteins in the body are degraded by autophagy to produce amino acids [40,41]. Amino acid accumulation via autophagy could explain the differential amino acid levels in the serum of WT and KO mice under fasting in this study [42]. On the other hand, due to the decreased transaminase activity caused by liver damage in GPRC6A mice, amino acids, especially glutamate, could not be transformed by transaminase. Accordingly, Glu in the serum of KO mice was significantly higher than in that of WT mice, and the deletion of GPRC6A led to high levels of UA in the serum of mice, possibly due to the elevated NH3L or disturbance of ornithine circulation [43,44]. In a KEGG pathway analysis, we found that the phenylalanine metabolic pathway was the most highly affected metabolic pathway after GPRC6A knockout. Under dietary protein and amino acid restrictions, the loss of function of GPRC6A would result in significant differences in purine metabolism in mice.

## 5. Conclusions

GPRC6A plays a crucial role in metabolism, mainly in lipid and amino acid metabolism; these processes may mainly be affected by liver function. The loss of GPRC6A affected the secretion of hepatic bile acids, thus affecting the metabolism of unsaturated fatty acids. It may also affect the metabolism of amino acids in mice during fasting. However, as an amino acid receptor, there are few reports on the effects of GPRC6A on amino acid metabolism in mice, and this issue should be a focus of future studies.

## Figures and Tables

**Table 1:** Diet formulations with different protein levels.

Ingredient (% by Weight)	10% Protein (LP)	20% Protein (NP)
Corn Sugar	46.06	39.75
Casein Lactic	10.00	20.00
Granular Sugar	11.74	10.00
Dextrin	15.30	13.20
Solka Floc-40	5.00	5.00
AIN-93 Mineral Mix	3.50	3.50
AIN-93 Vitamin Mix	1.00	1.00
l-Cystine	0.15	0.30
Choline Bitartrate	0.25	0.25
Soy Oil	7.00	7.00
Sum	100	100

Note: LP (Low protein level), NP (Normal protein level).

**Table 2:** Effect of the loss of GPRC6A on serum free amino acids in mice fed different protein levels.

AAs (μM)	WT–NP	KO–NP	WT–LP	KO–LP	*p*-Value	
					Genotype	Protein
His	60.5 ± 1.8	59.3 ± 3	65 ± 2.6	62.2 ± 2.7	0.432	0.152
Arg	114.6 ± 2.6	140.2 ± 3.9 **	146.9 ± 7.8	145.9 ± 7.5	0.059	0.005
Asn	51.1 ± 2	62 ± 5.2	55.1 ± 2.6	60.5 ± 1.4	0.013	0.693
Gln	480.8 ± 10.9	447.3 ± 19	556.8 ± 7.8	463 ± 8.7 ***	<0.001	0.001
Ser	102.3 ± 4.4	129.3 ± 5.1	136.9 ± 4.6	129.7 ± 3.1	0.166	0.019
Gly	249.2 ± 11.1	267.3 ± 8.5	281.8 ± 13	263.1 ± 8.1	0.979	0.192
Asp	12.2 ± 1	14 ± 0.9	11.7 ± 0.8	11.5 ± 0.7	0.366	0.087
Glu	50.2 ± 1.8	66.6 ± 3.5 **	52.8 ± 2.7	56.1 ± 1.5	<0.001	0.124
Thr	149.4 ± 6.5	175.1 ± 9.3 *	186.2 ± 7.6	194.6 ± 6.9	0.03	0.001
Ala	285.5 ± 5.5	344 ± 30.6	377.3 ± 21.7	321.7 ± 8.2 *	0.942	0.102
Pro	76.1 ± 4	96.7 ± 10.7	88.4 ± 3.2	93.7 ± 3.5	0.025	0.407
Lys	266.5 ± 12.5	301.3 ± 15.6	348.4 ± 16.1	325 ± 8.4	0.673	<0.001
Cys	2.1 ± 0.3	1.7 ± 0.1	1.7 ± 0.1	1.9 ± 0.2	0.79	0.61
Tyr	51.1 ± 2	79 ± 4.5 ***	50.5 ± 2.2	64.2 ± 2 ***	<0.001	0.009
Met	50.6 ± 1.9	63 ± 3.4 **	58 ± 1.5	57.3 ± 1.2	0.006	0.673
Val	212.1 ± 6.8	198.2 ± 9.6	188.2 ± 9.5	209.5 ± 7.4	0.665	0.462
Ile	96.3 ± 2.9	86.3 ± 5	83.9 ± 3.6	94.6 ± 2.6	0.927	0.593
Leu	170.6 ± 6.5	148.9 ± 7.5	150.1 ± 7.7	159.7 ± 8.6	0.435	0.531
Phe	78.2 ± 1.7	79 ± 3.3	72.7 ± 1.8	78.9 ± 2.9	0.173	0.263
Trp	70 ± 3	90.2 ± 3.5 ***	90.5 ± 4.6	89.3 ± 5.3	0.033	0.027

Note: Serum samples were collected after mice were fasted for 8 h. Asterisks indicate a significant difference between WT and KO mice with the same dietary protein level. * *p* < 0.05, ** *p* < 0.01, *** *p* < 0.001, *n =* 8–11.

## Data Availability

The data presented in this study are available on request from the corresponding author. The data are not publicly available due to privacy.

## Supplementary Materials

The following supporting information can be downloaded at: https://www.mdpi.com/article/10.3390/metabo12090776/s1. Figure S1: The effects of function loss of GPRC6A on mice fed with different dietary protein levels; Figure S2: Effect of function loss of GPRC6A on serum free amino acids in mice aged 55 weeks; Figure S3: Three-dimensional PCA score plots of plasma metabolites in GPRC6A WT and KO mice fed with different dietary protein levels, Table S1: Key parameters of differential metabolite (WT–NP and KO–NP); Table S2: Key parameters of differential metabolite (WT–LP and KO–LP).
