# Supplementary material for: Untargeted Metabolomics Reveals the Function of GPRC6A in Amino Acid and Lipid Metabolism in Mice
Source: Metabolites. 2022 Aug 23;12(9):776. doi: 10.3390/metabo12090776 (PMC9502419; doi:10.3390/metabo12090776)
Supplement: Supplementary file 1 [file metabolites-12-00776-s001.zip › Table S1 and Table S2.pdf]

Supplementary materials

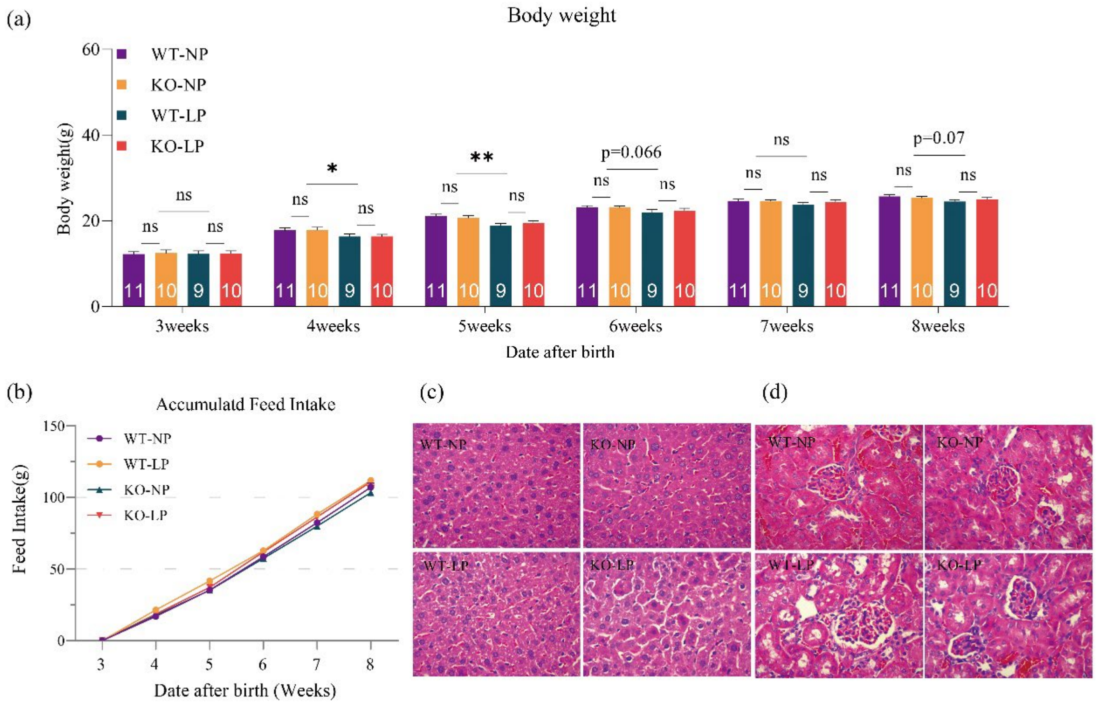

Figure S1. The effects of function loss of GPRC6A on mice fed with different dietary protein levels. (a) Body weight of GPRC6A mice fed with different protein levels during 3-8weeks. (b) Accumulated feed intake of mice during 0-8weeks. (c) Liver histomorphology of mice fed by different dietary protein levels at 8 weeks of age. (d) Kidney histomorphology of mice fed by different dietary protein levels at 8 weeks of age. The data were represented as mean± SEM, *p<0.05, **p<0.01, ns represents not significant, n=9-11. The magnification of the histomorphological images was 400×.

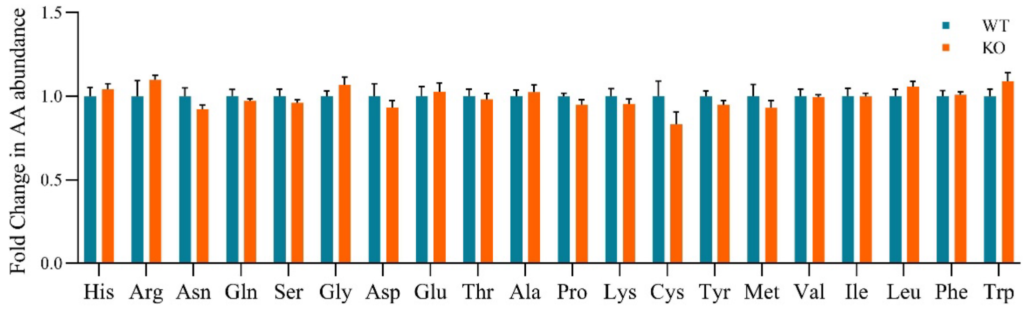

Figure S2. Effect of function loss of GPRC6a on serum free amino acids in mice aged 55 weeks, the data were normalized and were presented as mean ± SEM, n=7-8.

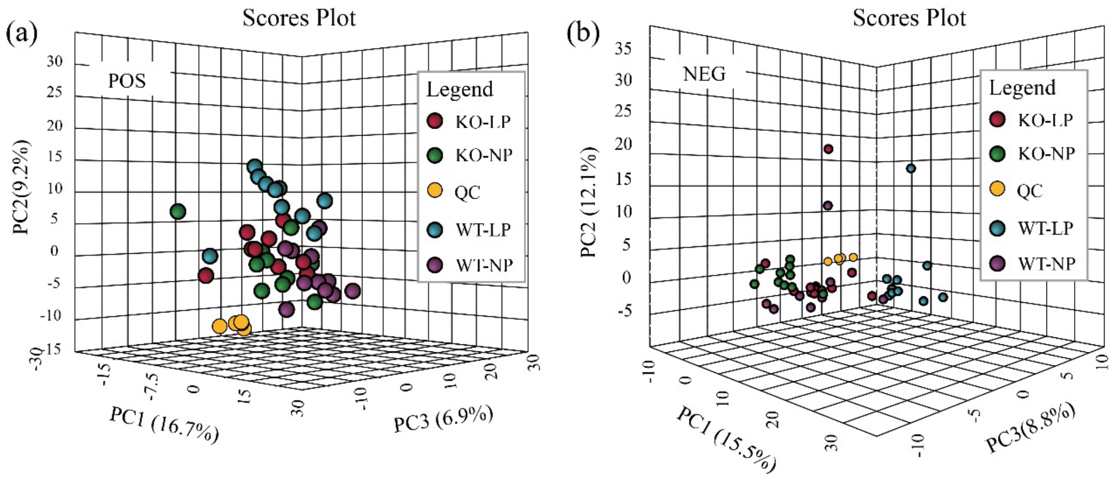

Figure S3. Three-dimensional PCA score plots of plasma metabolites in GPRC6A WT and KO mice fed with different dietary protein levels. (a) GPRC6A WT and KO mice under POS mode with QC. (b) (a) GPRC6A WT and KO mice under NEG mode with QC.

Table S1 Key parameters of differential metabolite (WT-NP&KO-NP)

mode	Metabolites	*p*-value	Fold Change	VIP
NEG	Stearidonic acid	3.75E-06	0.466	1.965
NEG	Alpha-Linolenic acid	9.87E-05	0.621	1.793
NEG	Deoxyadenosine	1.73E-04	0.387	1.622
NEG	Daidzin	1.95E-04	4.925	1.706
NEG	Palmitoleic acid	2.58E-04	0.581	1.534
NEG	3-Hydroxybutyric acid	4.91E-04	0.533	1.731
NEG	Hypoxanthine	5.21E-04	1.781	1.547
NEG	Xanthosine	5.59E-04	1.682	1.655
NEG	Citraconic acid	9.16E-04	4.397	1.581
NEG	Hypogeic acid	1.26E-03	0.571	1.506
NEG	2-Keto-3-deoxy-D-gluconic acid	1.67E-03	0.536	1.523
NEG	Bovinic acid	1.72E-03	0.654	1.552
NEG	13-OxoODE	1.76 E-03	0.6	1.59
NEG	12-HEPE	1.85E-03	0.569	1.456
NEG	4-Acetylbutyrate	1.87E-03	0.251	1.638
NEG	Kynurenic acid	2.024E-03	1.745	1.508
NEG	Ethyl dodecanoate	2.124E-03	0.632	1.535
NEG	Phenylacetylglycine	2.50E-03	1.572	1.449
NEG	Fumaric acid	2.72E-03	1.615	1.547
NEG	Sucrose	2.821E-03	2.692	1.264
NEG	Eicosadienoic acid	3.50E-03	0.663	1.383

NEG	3-Sulfinato-L-alaninate	3.51E-03	0.45	1.394
NEG	10E,12Z-Octadecadienoic acid	5.09E-03	0.452	1.265
NEG	Dihydrouracil	6.32E-03	0.616	1.45
NEG	Thromboxane B2	6.50E-03	0.579	1.324
NEG	Benzenebutanoic acid	7.21E-03	2.389	1.519
NEG	3-Methylcrotonylglycine	7.3E-03	0.101	1.411
NEG	Myristic acid	7.65E-03	0.209	1.41
NEG	2-Pyrocatechuic acid	8.03E-03	2.267	1.336
NEG	2-(3,4-dihydroxyphenyl)-3,5,7-trihydroxy-3,4-dihydro-2H-1-benzopyran-4-one	9.57E-03	2.327	1.477
NEG	Myristoleic acid	0.011	0.659	1.285
NEG	Oxoadipic acid	0.015	0.501	1.358
NEG	(R)-lipoic acid	0.015	1.582	1.343
NEG	Cortisone	0.017	1.729	1.152
NEG	S-Nitrosoglutathione	0.025	0.51	1.269
NEG	Hippuric acid	0.025	1.853	1.15
NEG	Prostaglandin E2	0.033	0.505	1.192
NEG	N-Formyl-L-methionine	0.036	0.55	1.254
NEG	Aldehydo-D-xylose	0.036	1.724	1.137
POS	Trimethylamine N-oxide	1.83E-05	1.821	2.515
POS	Zymonic acid	1.51E-04	5.343	2.378
POS	Xanthosine	4.9E-04	2.091	2.453
POS	1-Methylnicotinamide	5.62E-03	0.595	2.301
POS	Niacinamide	8.63E-04	0.641	2.345
POS	5-Ethyl-2,4-dimethyloxazole	1.20E-03	1.521	2.195
POS	L-Tyrosine	0.003351	1.78	1.999
POS	2-Phenylacetamide	3.50E-03	1.827	2.004
POS	Bitalin A 12-glucoside	0.007028	1.783	1.911
POS	Tetramethylsilane	7.34E-03	0.644	1.89
POS	4-Hydroxybenzaldehyde	7.86E-03	1.918	1.873
POS	Taurine	0.011	0.608	1.702
POS	Biliverdin	0.016	0.626	1.654
POS	Sphalleroside A	0.016	2.658	1.66
POS	N-Carbamoylsarcosine	0.017	0.664	1.737
POS	5-Aminoimidazole ribonucleotide	0.029	1.753	1.57
POS	PC(18:2(9Z,12Z)/14:1(9Z))	0.041	0.604	1.478

Table S2 Key parameters of differential metabolite (WT-LP&KO-LP)

mode	metabolites	*p*-value	Fold Change	VIP
NEG	6-trans-Leukotriene B4	1.6E-05	1.852	2.425
NEG	Xanthosine	5.23E-05	1.883	1.938

NEG	12-HETE	9.58E-05	1.996	2.388
NEG	20-Hydroxyeicosatetraenoic acid	2.96E-04	2.086	2.325
NEG	Hypoxanthine	6.63E-04	2.195	1.896
NEG	Uric acid	7.82E-04	1.868	1.954
NEG	13,14-Dihydro-15-keto PGF2a	1.04E-03	3.113	2.099
NEG	D-2,3-Dihydroxypropanoic acid	1.907E-03	0.658	1.718
NEG	12-HEPE	5.6E-03	1.846	2.061
NEG	Benzenebutanoic acid	6.77E-03	0.456	2.024
NEG	Perillic acid	6.88E-03	0.635	1.807
NEG	L-Gulonolactone	9.52E-03	0.656	1.528
NEG	12-Oxo-2,3-dinor-10,15-phytodienoic acid	9.56E-03	1.793	1.273
NEG	Inosine	9.74E-03	1.924	1.701
NEG	Thromboxane B2	0.014	2.153	1.544
NEG	Traumatic acid	0.017	2.491	1.831
NEG	10E,12Z-Octadecadienoic acid	0.019	2.705	1.396
NEG	13-OxoODE	0.024	1.633	1.448
NEG	4-Acetylbutyrate	0.030	6.387	1.691
NEG	Oxoadipic acid	0.032	0.549	1.371
NEG	Citraconic acid	0.033	0.619	1.286
NEG	(-)-Matairesinol	0.034	0.657	1.396
NEG	3-(3,4-Dimethoxyphenyl)-2-propenoic acid	0.036	1.526	1.464
POS	Zymonic acid	1.41E-06	13.823	2.577
POS	Xanthosine	2.57E-04	2.057	2.139
POS	Uralenneoside	1.13E-03	2.026	2.002
POS	trans-Hexadec-2-enoyl carnitine	2.43E-03	1.568	2.2
POS	Furcelleran	2.62E-03	0.602	1.022
POS	Nebularine	4.36E-03	2.018	1.583
POS	3-Acetamidobutanal	5.41E-03	1.725	1.48
POS	Clionasterol	6.20E-03	0.616	1.804
POS	Hexanoylglycine	0.011	1.753	1.763
POS	Isovalerylglutamic acid	0.028	0.609	1.96
POS	Terbutaline	0.030	2.367	1.611
POS	5-Deoxykievitol	0.037	2.071	1.652
POS	Histidinal	0.0423	0.586	1.946

R command for OPLS-DA and permutation

1. mSet<-InitDataObjects("conc", "stat", FALSE)
2. mSet<-Read.TextData(mSet, "Replacing_with_your_file_path", "colu", "disc");
3. mSet<-SanityCheckData(mSet)
4. mSet<-ReplaceMin(mSet);
5. mSet<-SanityCheckData(mSet)

6. mSet<-FilterVariable(mSet, "iqr", "F", 25)
7. mSet<-PreparePrenormData(mSet)
8. mSet<-Normalization(mSet, "SumNorm", "CrNorm", "AutoNorm", ratio=FALSE, ratioNum=20)
9. mSet<-PlotNormSummary(mSet, "norm_0_", "png", 72, width=NA)
10. mSet<-PlotSampleNormSummary(mSet, "snorm_0_", "png", 72, width=NA)
11. mSet<-OPLSR.Anal(mSet, reg=TRUE)
12. mSet<-PlotOPLS2DScore(mSet, "opls_score2d_0_", "png", 72, width=NA, 1,2,0.95,0,0)
13. mSet<-PlotOPLS.Splot(mSet, "opls_splot_0_", "all", "png", 72, width=NA);
14. mSet<-PlotOPLS.Imp(mSet, "opls_imp_0_", "png", 72, width=NA, "vip", "tscore", 15,FALSE)
15. mSet<-PlotOPLS.MDL(mSet, "opls_mdl_0_", "png", 72, width=NA)
16. mSet<-OPLSDA.Permut(mSet, 100)
17. mSet<-PlotOPLS.Permutation(mSet, "opls_perm_1_", "png", 72, width=NA)

R command for PCA

1. mSet<-InitDataObjects("conc", "stat", FALSE)
2. mSet<-Read.TextData(mSet, "Replacing_with_your_file_path", "colu", "disc");
3. mSet<-SanityCheckData(mSet)
4. mSet<-ReplaceMin(mSet);
5. mSet<-SanityCheckData(mSet)
6. mSet<-FilterVariable(mSet, "iqr", "F", 25)
7. mSet<-PreparePrenormData(mSet)
8. mSet<-Normalization(mSet, "SumNorm", "CrNorm", "AutoNorm", ratio=FALSE, ratioNum=20)
9. mSet<-PlotNormSummary(mSet, "norm_0_", "png", 72, width=NA)
10. mSet<-PlotSampleNormSummary(mSet, "snorm_0_", "png", 72, width=NA)
11. mSet<-PCA.Anal(mSet)
12. mSet<-PlotPCAPairSummary(mSet, "pca_pair_0_", "png", 72, width=NA, 5)
13. mSet<-PlotPCAScree(mSet, "pca_scree_0_", "png", 72, width=NA, 5)
14. mSet<-PlotPCA2DScore(mSet, "pca_score2d_0_", "png", 72, width=NA, 1,2,0.95,0,0)
15. mSet<-PlotPCALoading(mSet, "pca_loading_0_", "png", 72, width=NA, 1,2);
16. mSet<-PlotPCABiplot(mSet, "pca_biplot_0_", "png", 72, width=NA, 1,2)
17. mSet<-PlotPCA3DLoading(mSet, "pca_loading3d_0_", "json", 1,2,3)

R command for t-Test

1. mSet<-InitDataObjects("conc", "stat", FALSE)
2. mSet<-Read.TextData(mSet, "Replacing_with_your_file_path", "colu", "disc");
3. mSet<-SanityCheckData(mSet)
4. mSet<-ReplaceMin(mSet);
5. mSet<-SanityCheckData(mSet)
6. mSet<-FilterVariable(mSet, "iqr", "F", 25)
7. mSet<-PreparePrenormData(mSet)
8. mSet<-Normalization(mSet, "SumNorm", "CrNorm", "AutoNorm", ratio=FALSE, ratioNum=20)
9. mSet<-PlotNormSummary(mSet, "norm_0_", "png", 72, width=NA)
10. mSet<-PlotSampleNormSummary(mSet, "snorm_0_", "png", 72, width=NA)
11. mSet<-Ttests.Anal(mSet, F, 0.05, FALSE, TRUE, "fdr", FALSE)
12. mSet<-PlotTT(mSet, "tt_0_", "png", 72, width=NA)
